# Identification of signal-based gait features and blood analytes associated with stroke status and walking speed in mild acute ischemic stroke

**DOI:** 10.1186/s12883-026-05057-3

**Published:** 2026-06-12

**Authors:** Meryem Şahin Erdoğan, Mete Özgün, Dilaver Kaya, Otar Akanyeti, Hale Saybaşılı

**Affiliations:** 1 Institute of Biomedical Engineering, Boğaziçi University, Uskudar, Istanbul, Turkey; 2 Neurology, Acıbadem Mehmet Ali Aydınlar University, Atasehir, Istanbul, Turkey; 3 Department of Computer Science, Aberystwyth University, Aberystwyth, United Kingdom

**Keywords:** Blood analytes, Gait analysis, Mild acute ischemic stroke, Nomogram, Walking speed

## Abstract

**Background:**

Assessment of mild acute ischemic stroke (mAIS) can be difficult, especially in regions where access to neuroimaging is limited. Integrating routine blood analyses with wearable gait assessments may aid in the identification of mAIS and estimation of functional outcomes. In this pilot study, we focus on identifying candidate gait and blood analytes associated with stroke status and functional outcomes in mAIS.

**Methods:**

Video, smartphone, and blood data were collected from 22 mAIS patients and nine healthy controls. Ridge-penalized logistic regression nomograms were developed; one to estimate the stroke status using gait, and a second one to estimate walking speed of patients using blood analytes.

**Results:**

Our gait-based nomogram identified mAIS with an area under the curve (AUC) of 0.878, while blood-based nomogram estimated walking speed with an AUC of 0.906 (both optimism-corrected). Identified gait features for stroke status included lower xgyr_energy, xgyr_max, xgyr_std, zgyr_energy and zacc_rms, and higher zgyr_iqr, while higher age, blood urea nitrogen (BUN), and lower estimated glomerular filtration rate (eGFR) and mean corpuscular hemoglobin concentration (MCHC) were associated with slower walking.

**Conclusions:**

We show the feasibility of integrating gait statistics with blood analytes to identify mAIS and estimate walking speed. The nomograms showed strong discrimination and calibration within our small cohort. These findings point to the potential of multimodal signal-based features in stroke detection and rehabilitation planning, particularly in low-resource settings.

**Supplementary Information:**

The online version contains supplementary material available at 10.1186/s12883-026-05057-3.

## Introduction

Stroke is a markedly heterogeneous condition, creating substantial challenges in the assessment of mild acute ischemic stroke (mAIS) and in the estimation of functional outcome, which is essential for clinical decision-making and long-term patient planning.

In settings without advanced healthcare technologies, it is essential to extract clinically meaningful information from widely accessible and inexpensive modalities. Routine blood analysis, such as complete blood count and electrolyte levels, is available in most clinics. However, no established blood biomarker currently exists for mAIS.

Recent studies suggest prognostic value in several routine blood analytes. Elevated C-reactive protein (CRP) is associated with worse long-term outcomes [1] and kidney function markers, estimated glomerular filtration rate (eGFR) [2] and blood urea nitrogen (BUN) [3], also correlate with ischemic stroke prognosis, with poorer renal function being associated with poorer outcomes.

Gait impairment and reduced walking affect roughly half of patients in the acute stage of stroke [4]. Walking speed is an important indicator of post-stroke disability and morbidity [5–8]. Although objective gait assessment traditionally requires specialized motion capture systems, recent advances in affordable wearable sensors have enabled the measurement of parameters such as walking speed, step asymmetry and cadence [9–11]. Clinicians can better estimate functional outcomes and tailor personalized rehabilitation techniques by integrating these metrics with blood analyses and clinical assessment. Such a multimodal approach supported by advanced statistical methods could uncover clinically meaningful insights, given the heterogeneous nature of stroke [12].

mAIS is generally defined as National Institutes of Health Stroke Scale (NIHSS) ≤ 5 at presentation [13, 14]. A prior study demonstrated the feasibility of using wearable technology to monitor daily activities after mild stroke [15] yet, to our knowledge, no studies combined statistical gait features with blood analytes to identify stroke status or estimate functional clinical outcomes. Even though the presence of stroke and walking speed can be directly assessed in most clinical settings, access to neuroimaging and standardized gait testing may be limited in remote or low-resource environments. Utilizing accessible and inexpensive equipment and routine blood tests to extract features would be particularly valuable in settings where access to advanced medical imaging technologies is limited, making the diagnosis of mAIS more complicated.

In this study, we combine signal-based gait features and blood analytes to develop two nomograms: one for estimating mAIS status and another for estimating walking speed outcome. Nomograms were chosen for their simplicity, interpretability and suitability for clinical use. Also, they provide the user with quantitative probabilities rather than observations. The study cohort was intentionally restricted to mAIS patients, an underrepresented group that poses unique diagnostic and prognostic challenges.

## Methods

### Patients

The study protocol was approved by Acıbadem University and Acıbadem Healthcare Institutions Medical Research Ethics Committee (ATADEK-2021/21–106). This prospective observational exploratory study had 31 participants including 22 mAIS patients and nine healthy controls. The sample size was determined by the number of eligible patients who presented during the recruitment window. Data was collected between April 2023 and March 2025 in Acıbadem Altunizade Hospital. All identifying participant information was kept in a locked room and in a locked drawer. Healthy controls were selected from the hospital staff, and the family and friends of the patients who did not have prior ischemic stroke. We have included adult patients with mAIS (NIHSS ≤ 5), who are ambulatory within 2 days of stroke. We have excluded patients with bilateral ischemic stroke, unambulatory patients, patients unable to comprehend verbal directions and patients with conditions impairing gait other than ischemic stroke. This study is reported in accordance with the Transparent Reporting of a Multivariable Prediction Model for Individual Prognosis or Diagnosis (TRIPOD) checklist for exploratory model development with internal validation [16].

### Gait analysis

Gait analysis tests were performed within two days of the ischemic stroke, in the presence of a neurologist and a physiotherapist. A smartphone (Samsung Galaxy S20 FE) was placed on the back of the patient on the L3 vertebrae, and the accelerometer and gyroscope within the device were utilized with MATLAB Mobile to track gait. A GoPro Hero 8 camera was used to monitor the walking performance tests which were 10-meter walking and Timed-up-and-go (TUG). The tests were performed twice to ensure consistency and allow for subject acclimation to the procedure. Participants were instructed to walk at their preferred speed and were permitted to use walking aids if necessary; however, patients were excluded if they could not complete the tests due to fatigue or pain. To ensure stable performance, signals from the second trial of the 10-meter walking test were selected for feature extraction. Signals were collected from the accelerometer and gyroscope embedded in the smartphone (100 Hz sampling frequency) during the second trial of the 10-meter walking. Walking bouts were manually segmented from the continuous smartphone recordings by synchronizing signal timestamps with the camera footage. For the 10-meter walking test, subjects were instructed to stop upon reaching the opposite wall to avoid the inclusion of turning points. Signal preprocessing involved defining the X, Y, and Z axes as vertical, medio-lateral, and antero-posterior, respectively. To eliminate high-frequency noise, a 4th-order zero-phase Butterworth low-pass filter with a 5 Hz cutoff was applied. To standardize signal amplitude z-score normalization was applied.

Gait features were extracted using a dual-modality approach: some gait features were derived from the camera footage, while signal-based gait statistics were extracted globally over the entire identified walking bout from the smartphone inertial signals. Standard statistical features including mean, median, standard deviation (std), mean absolute deviation (mad), interquartile range (iqr), min, max, range, skewness, kurtosis, root mean square (rms), energy, entropy and number of zero crosses were computed for all three axes of both acceleration and angular velocity using Python’s SciPy library [17]. These statistical features were selected to capture global signal magnitude and variability, reflect movement signatures, changes in propulsion and dynamic balance characteristic of mAIS using accessible equipment and straightforward calculations, rather than capturing validated gait constructs. Full list of gait features is given in Supplementary Table 1.

Also, we have extracted five relevant gait information from the camera footage: left foot stance ratio (CamLeftStance), right foot stance ratio (CamRightStance), mean stance ratio (CamMeanStance), TUG time (s) (CamTUG) and walking speed (m/s) (CamWalkingSpeed). Stance, which makes up about around 60% of a complete gait cycle [18], is calculated as;

$$Stance\:=\:\frac{Stance\:duration}{Gait\:cycle\:duration}\times\:100$$

TUG time was calculated over two trials, and the start and finish times were recorded as the time subject started to get up from the chair by lowering their trunk forward and the time subject leaned back in the chair. Lastly, walking speed was calculated over the 5-meter walking path that was marked by red tape and recorded on the camera. Start and finish times were recorded as the times when subjects’ trunk were over the red tape.

### Clinical evaluations and blood analyses

Physician’s reports on the demographic information, preexisting conditions, current diagnoses of the patients and NIHSS scores were recorded. NIHSS evaluates the impairment levels post-stroke. The scale ranges between zero and 42, with higher scores implying higher impairment. We have also obtained blood test results of all subjects which generally included total blood count and electrolyte analyses. Aside from the medically necessary blood draws and blood analyses, we have not performed additional blood draws or tests either on patients or control subjects.

The blood analyses tests included a comprehensive list of hematological and biochemical markers: Absolute basophil count (Baso#), Absolute eosinophil count (Eos#), Absolute lymphocyte count (Lymph#), Absolute monocyte Count (Mono#), Absolute Neutrophil Count (Neut#), along with their respective percentages (Baso%, Eos%, Lymph%, Mono%, Neut%), Red blood cell count (RBC), White blood cell count (WBC), Hemoglobin (HGB), Hematocrit (HCT), Mean corpuscular volume (MCV), Mean corpuscular hemoglobin (MCH), Mean corpuscular hemoglobin concentration (MCHC), Red cell distribution width-coefficient of variation (RDW-CV), and Red cell distribution width-standard deviation (RDW-SD). Platelet-related parameters included Platelet count (PLT), Mean platelet volume (MPV), Platelet distribution width (PDW), and Platelet large cell ratio (P-LCR). Also, Neutrophil-to-lymphocyte ratio (NLR) and Macrocyte ratio (Macro-R) were recorded. Evaluated renal and inflammatory markers and electrolytes were Blood urea nitrogen (BUN), Urea, Creatinine (Cr), Estimated glomerular filtration rate (eGFR), Procalcitonin (PCT), Sodium (Na) and Potassium (K).

**Fig. 1:**
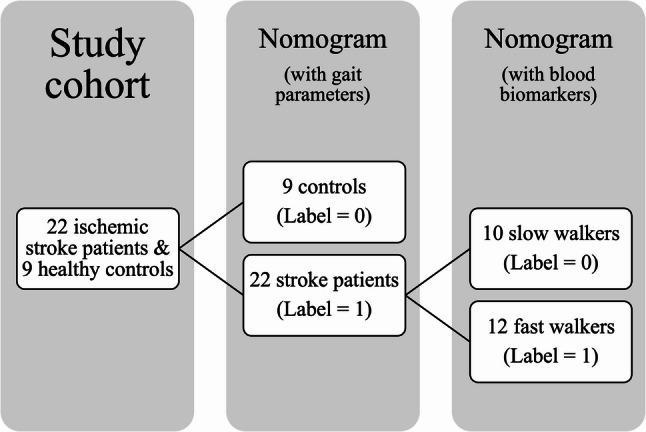
Overview of the study workflow showing the number of subjects included for nomogram development

Participants were classified according to their clinical evaluations and gait features. A comma-separated file, including demographic information, blood analysis and gait analysis was prepared. We first evaluated data from each modality to see if there are any statistically significant differences between groups with Mann-Whitney test. Statistical results were reported as: mean ± sd. The workflow of the study is shown in Fig. 1.

### Nomogram

We developed two nomograms in order to estimate (i) control (*n* = 9, label = 0) vs. stroke (*n* = 22, label = 1) groups of participants based on gait features, and (ii) walking speed status of stroke patients, as slow-walkers (*n* = 10, label = 0) vs. fast-walkers (*n* = 12, label = 1), based on age and blood analytes.

NIHSS and sex variables we excluded to be able to develop solely gait and blood-based models, even though they were not retained in the final models. Missing blood analyte values were imputed using median imputation since it is a robust and simple imputation method, even though it may result in a slight reduction in variation in very small datasets [19, 20]. To assess the robustness of this approach, we performed a leave-one-out sensitivity analysis on key clinical predictors, calculating the ΔAUCs for each variable. Additionally, we compared the performance of the full model against a reduced feature set comprising of the most complete analytes to evaluate potential bias introduced by the imputation of analytes with higher missingness. Then, features were screened one by one using univariate logistic regression (likelihood-ratio, χ^2^), and the K_top_ features with the smallest *P*-values were carried forward, and outcome-free redundancy pruning was applied. Afterwards, a multivariable logistic regression model with an L2-regularized ridge penalty (λ = 1) was fitted to address multicollinearity and reduce overfitting in our small cohort. This shrinks the regression coefficients toward zero, which reduces model variance and improves the model’s generalizability. A fixed penalty was preferred over cross-validation to stabilize coefficients and prevent overfitting given the limited sample size.

The resulting regression coefficients were then used to construct the nomogram, in which each predictive feature receives points proportional to its effect on the outcome. Discrimination was summarized using the area under the curve (AUC) values, and also 95% confidence intervals (CI) obtained by bootstrapping (B = 1000). Calibration was assessed with a bootstrap calibration curve (B = 500). Internal validation used bootstrap resampling (B = 500) to estimate optimism in the calculated AUC. To decide the K_top_, we re-ran the full pipeline across candidate values (3–12), recording AUC values for each, and chose the K_top_ value that maximized the corrected AUC while maintaining a lower number of features (Supplementary Figs. 1 and 2). Analyses were conducted in R (rms and Hmisc packages).

## Results

### Patient characteristics

Patients were in the acute stage of stroke, with time-since-stroke being 1.2 ± 1.2 days. There were no statistically significant differences between stroke (range = 29–90) and control (range = 38–78) groups in terms of sex (*P* = 0.566) and age (*P* = 0.058). All participants were able to complete both 10-meter walk and TUG tests. Since the patients were ambulatory during the acute stage of stroke, our cohort was restricted to individuals with mild impairment.

### Gait analysis

The statistical analyses of the gait statistics extracted from the smartphone and the camera is shown in Table 1 (Full list of gait features and their statistical comparisons can be found in Supplementary Table 1). To enable analysis of blood analytes that were unavailable for the control subjects, stroke patients were stratified according to CamWalkingSpeed: slow walkers (CamWalkingSpeed < 0.60, label: 0) and fast walkers (CamWalkingSpeed ≥ 0.60 m/s, label: 1). 0.6 m/s was selected since it was indicated to be a cutoff for community-dwelling abilities [21] and a higher risk of mortality [22].

**Table 1:** Comparison of statistically different gait features between control and stroke groups. Data is reported as mean±standard deviation (*: *P* < 0.05, **: *P* < 0.01). CamLeftStance: Left foot stance ratio calculated using the camera footage, CamWalkingSpeed: Walking speed calculated from the camera footage. x, y and z at the beginning of the feature names represent x-, y- and z-axes. gyr stands for gyroscope, while acc represents accelerometer. The parts after the underscore represent statistical features of the signals collected from the gyroscope or the accelerometer; iqr: interquartile range, mad: mean absolute deviation, max: maximum, rms: root mean square, std: standard deviation, min: minimum

Features	Control (*n* = 9) (mean ± sd)	Stroke (*n* = 22) (mean ± sd)	*P*-value	Features	Control (*n* = 9) (mean ± sd)	Stroke (*n* = 22) (mean ± sd)	*P*-value
CamLeftStance*	66.27 ± 3.62	72.48 ± 10.2	0.039	ygyr_iqr*	0.26 ± 0.03	0.23 ± 0.06	0.043
CamWalkingSpeed*	1.02 ± 0.26	0.71 ± 0.42	0.035	zacc_mad*	0.97 ± 0.31	0.75 ± 0.34	0.048
xacc_entropy*	3.33 ± 0.2	3.08 ± 0.3	0.018	zacc_rms**	2.52 ± 0.86	1.55 ± 0.86	0.007
xacc_iqr*	1.7 ± 0.85	1.22 ± 1.02	0.048	zacc_std*	1.25 ± 0.42	0.94 ± 0.44	0.039
xacc_kurtosis*	1.76 ± 1.59	4.09 ± 2.94	0.043	zgyr_energy**	135.11 ± 67.36	71.62 ± 47.93	0.007
xgyr_energy**	664.5 ± 226.03	356.83 ± 214.75	0.003	zgyr_iqr*	0.37 ± 0.14	0.23 ± 0.11	0.031
xgyr_mad*	0.43 ± 0.12	0.31 ± 0.13	0.018	zgyr_mad*	0.22 ± 0.07	0.15 ± 0.07	0.018
xgyr_max**	2.41 ± 0.92	1.37 ± 0.75	0.007	zgyr_max*	0.9 ± 0.28	0.64 ± 0.27	0.018
xgyr_mean**	0.14 ± 0.15	0.03 ± 0.1	0.004	zgyr_min*	-0.84 ± 0.34	-0.6 ± 0.25	0.025
xgyr_range*	3.49 ± 1.13	2.47 ± 0.91	0.022	zgyr_range*	1.74 ± 0.6	1.24 ± 0.52	0.02
xgyr_rms**	0.66 ± 0.2	0.43 ± 0.18	0.007	zgyr_rms*	0.29 ± 0.09	0.19 ± 0.09	0.016
xgyr_skewness*	1.35 ± 1.4	0.33 ± 1.29	0.022	zgyr_std*	0.29 ± 0.09	0.19 ± 0.09	0.02
xgyr_std**	0.63 ± 0.2	0.42 ± 0.17	0.007				

### Clinical evaluations and blood analyses

NIHSS scores ranged from one to four with a mean score of 2. Blood tests were performed only on stroke patients and not on controls. Since there was no single test performed on all patients, data imputation was warranted to include all patients into the following analyses. Median imputer was used for all features where the number of non-existent samples is less than 40% the number of samples. After we imputed missing data using median imputation, we ended up with 32 blood analysis features. The descriptive statistics of those blood test results (including median imputed data) are shown in Table 2 (Full list of blood analytes and their statistical comparisons can be found in Supplementary Table 2). Notably, slow walking stroke patients tended to have higher Age, Baso#, Eos#, Mono#, Neut#, BUN, Cr, Urea values, and lower eGFR, MCH, MCHC and MCV values compared to fast walkers.

**Table 2:** Comparison of statistically different analytes between slow walkers and fast walkers. Data is reported as mean±standard deviation (*: *P* < 0.05, **: *P* < 0.01). MCH: Mean corpuscular hemoglobin, MCHC: Mean corpuscular hemoglobin concentration, MCV: Mean corpuscular volume

Features	Slow walkers (*n* = 10) (mean ± sd)	Fast walkers (*n* = 12) (mean ± sd)	*P*-value
Age**	78.8 ± 4.96	59.67 ± 18.38	0.01
MCH*	28.14 ± 0.97	29.26 ± 2.19	0.029
MCHC*	32.73 ± 0.8	33.47 ± 0.82	0.039
MCV*	86.14 ± 1.87	87.79 ± 4.83	0.043

### Nomogram

**Fig. 2:**
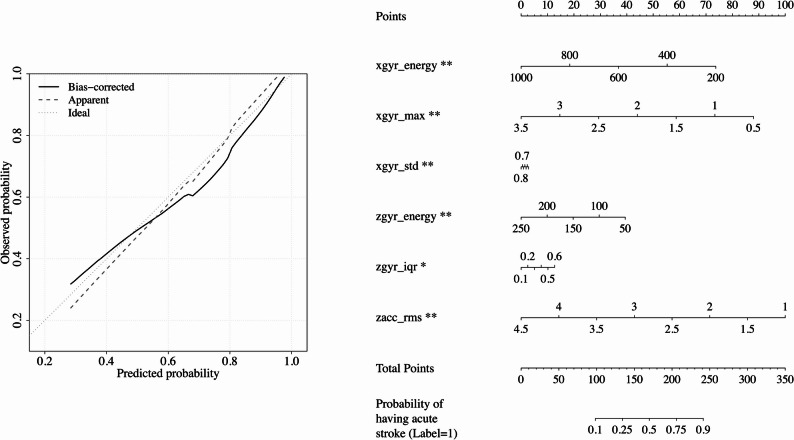
**A** Bootstrap bias-corrected calibration curve (B = 500) for the gait statistics-based nomogram for estimating the probabilities of participants being in the control vs. stroke groups. **B** Developed nomogram based on selected gait features. x and z at the beginning of the feature names represent x- and z-axes. gyr stands for gyroscope, while acc represents accelerometer. The parts after the underscore represent statistical features of the signals collected from the gyroscope or the accelerometer; max: maximum, std: standard deviation, iqr: interquartile range, rms: root mean square. (*: *P* < 0.05, **: *P* < 0.01)

The ridge-penalized logistic regression model estimating stroke status using gait features included six gait metrics: xgyr_energy, xgyr_max, xgyr_std, zgyr_energy, zgyr_iqr, zacc_rms. xgyr_rms, zgyr_rms were excluded from the nomogram development as redundant based on outcome-free redundancy analysis (R^2^ = 0.95). Calibration curve and developed nomogram are shown in Fig. 2. The calibration curve demonstrated strong agreement between predicted and observed probabilities. The bias-corrected estimate was adjacent to the 45-degree ideal diagonal, confirming that the model provides accurate risk stratification with very small systematic bias. Lower values in most metrics, except zgyr_iqr corresponded to higher estimated probabilities of having acute stroke.

In the fitted logistic regression model, xgyr_max (odds ratio (OR):0.41, 95% CI:0.12–1.42) and zacc_rms (OR:0.42, 95% CI:0.12–1.40) were associated with reduced odds of acute stroke. zgyr_iqr (OR:2.18, 95% CI:0.01–5.2 × 10⁴) showed a weak positive trend.

**Fig. 3:**
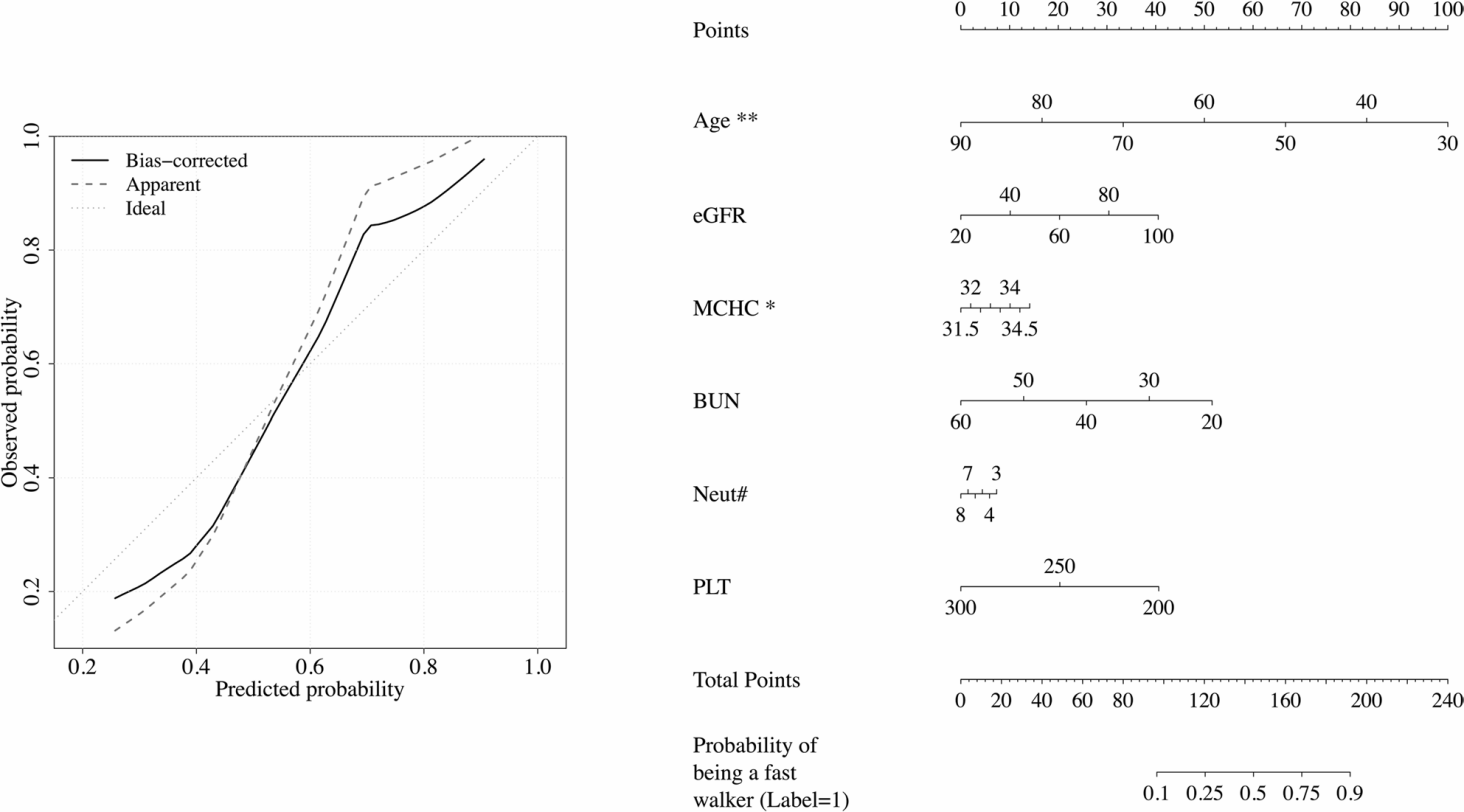
**A** Bootstrap bias-corrected calibration curve (B = 500) for the blood analyte-based nomogram for estimating the probabilities of participants being in the fast walker vs. slow walker groups. **B** Developed nomogram based on selected blood analytes. eGFR: estimated Glomerular Filtration Rate, MCHC: Mean Corpuscular Hemoglobin Concentration, BUN: Blood Urea Nitrogen, Neut#: Neutrophil count, PLT: Platelet count (*: *P* < 0.05, **: *P* < 0.01)

The walking speed model used six blood analytes: Age, eGFR, MCHC, BUN, Neut# and PLT, while Urea was excluded as redundant. Missing value percentages for the selected blood tests were; 27% for eGFR, 36% for MCHC, 32% for BUN, 36% for Neut# and 36% for PLT which represent moderate missingness, which calls for a sensitivity analysis. The sensitivity analysis revealed that the model was most sensitive to the exclusion of PLT and BUN, which contributed 0.025 and 0.017 to the total AUC, respectively. The inclusion of eGFR, MCHC, and Neut# showed minimal impact on the overall discriminative performance with ΔAUC < 0.01. A reduced feature set analysis using only the most complete analytes Age, eGFR, and Cr revealed that the model using these features maintained strong discriminative performance with an optimism-corrected AUC of 0.800, compared to 0.906 for the full model. Crucially, the regression coefficients for age and renal function markers remained stable in both direction and magnitude across both models (e.g., eGFR; Full model = 0.023 vs. Reduced model = 0.029). These analyses suggest that median imputation did not substantially alter the main findings. Calibration curve and developed nomogram are shown in Fig. 3. The calibration curve showed a high degree of fit, with a slight deviation in the tails of the probability distribution. Despite minor overestimation of risk in the lower deciles, the bias-corrected curve indicates that the model remained well-calibrated. In the logistic regression model, some features displayed directional trends but none of these had significant associations with faster walking. Higher age was associated with slightly lower odds of faster walking (OR:0.93, 95% CI:0.85–1.00). Similarly, higher BUN (OR:0.94, 95% CI:0.83–1.07) and Neut# (OR:0.93, 95% CI:0.41–2.13) were associated with lower odds of faster walking. Less prominently, MCHC exhibited an unclear trend (OR:1.20, 95% CI:0.28–5.14). These findings suggest that no single analyte dominated the model; instead, several contributed modestly. Internal validation was performed using bootstrap resampling (B = 500) for both nomograms. Table 3 lists accuracy and validation metrics of both nomograms.

**Table 3:** Accuracy and validation metrics of both developed nomograms. AUC: Area under the curve

	Control vs. stroke nomogram	Fast vs. slow walking nomogram
AUC	0.924	0.958
95% Confidence interval of AUC	0.81-1.00	0.86-1.00
Optimism value	0.047	0.052
Optimism-corrected AUC	0.878	0.906
Brier score	0.10	0.10
Calibration slope	1.01	1.11
Calibration intercept	-0.01	-0.02

### Nomogram estimation calculation

To enable individual-level estimation, we report the full logistic regression models used to estimate the stroke status and faster walking. The final ridge penalized model intercept for having mAIS was 6.274, and the corresponding regression coefficients were: xgyr_energy (β = -0.003), xgyr_max (β = -0.902), xgyr_std (β = -0.144), zgyr_energy (β = -0.006), zgyr_iqr (β = 0.780), and zacc_rms (β = -0.879). The estimation equation is:

$$\begin{aligned} \:logit\left(P\right)\:=&\:6.274+\left(-0.003\:\times\:\mathrm{x}\mathrm{g}\mathrm{y}\mathrm{r}\_\mathrm{e}\mathrm{n}\mathrm{e}\mathrm{r}\mathrm{g}\mathrm{y}\right)+\left(-0.902\times\:\mathrm{x}\mathrm{g}\mathrm{y}\mathrm{r}\_\mathrm{m}\mathrm{a}\mathrm{x}\right)\\&+\left(-0.144\times\:\mathrm{x}\mathrm{g}\mathrm{y}\mathrm{r}\_\mathrm{s}\mathrm{t}\mathrm{d}\right)+\left(-0.006\times\:\:\mathrm{z}\mathrm{g}\mathrm{y}\mathrm{r}\_\mathrm{e}\mathrm{n}\mathrm{e}\mathrm{r}\mathrm{g}\mathrm{y}\right)\\&+(0.780\times\:\mathrm{z}\mathrm{g}\mathrm{y}\mathrm{r}\_\mathrm{i}\mathrm{q}\mathrm{r})+(-0.879\times\:\mathrm{z}\mathrm{a}\mathrm{c}\mathrm{c}\_\mathrm{r}\mathrm{m}\mathrm{s}) \end{aligned}$$

where P represents the estimated probability of having mAIS.

The final ridge penalized model intercept for faster walking was 3.666, and the corresponding regression coefficients were: Age (β = -0.077), eGFR (β = 0.023), MCHC (β = 0.186), BUN (β = -0.059), Neut# (β = -0.068), and PLT (β = -0.019). The estimation equation is:

$$\begin{aligned} \:logit\left(P\right)\:=\:&3.666+\left(-0.077\:\times\:\mathrm{A}\mathrm{g}\mathrm{e}\right)+\left(0.023\times\:\mathrm{e}\mathrm{G}\mathrm{F}\mathrm{R}\right)\\&+\left(0.186\times\:\mathrm{M}\mathrm{C}\mathrm{H}\mathrm{C}\right)+\left(-0.059\times\:\:\mathrm{B}\mathrm{U}\mathrm{N}\right)\\&+(-0.068\times\:\mathrm{N}\mathrm{e}\mathrm{u}\mathrm{t}\#)+(-0.019\times\:\mathrm{P}\mathrm{L}\mathrm{T}) \end{aligned}$$

where P represents the estimated probability of faster walking.

Individual estimations can be obtained from both models using:

$$\:P\:=\:\frac{1}{(1+\mathrm{e}\mathrm{x}\mathrm{p}(-logit\left(P\right)\left)\right)}$$

## Discussion

This study investigated the associations of signal-based gait features and blood analytes of mAIS patients in relation to stroke status and functional outcome measured by walking speed. We developed two discriminative nomograms to estimate the stroke status and walking speed. The most informative gait statistics in our models were extracted from X- and Z-axis accelerometer and gyroscope signals, which represent statistical descriptors of signal magnitude and variability rather than direct biomechanical measures. In our cohort, these features were generally lower in stroke patients, suggesting reduced overall movement intensity and variability during walking. The trends observed in our exploratory study are consistent with the expected impaired motor performance following stroke. Nonetheless, the current analysis does not directly quantify specific biomechanical constructs or gait parameters. Our results showed that walking speed decreases in stroke patients and stance ratio remain around 60% for healthy controls, as expected [18, 23]. However, the literature is inconsistent on the relationship of clinical outcomes and MCHC, MCH and MCV levels [24, 25]. Nonetheless, lower MCHC levels were found to be associated with worse outcomes after cardiovascular events [26, 27].

In our cohort, reduced eGFR and elevated BUN levels are associated with slower walking speed, suggesting that impaired renal function may contribute to reduced physical performance. Prior studies consistently report that renal impairments are associated with poorer clinical outcomes [2, 3]. Beyond overall prognosis, renal dysfunction is linked to metabolic imbalance and reduced exercise tolerance [28], which may affect mobility and walking capacity. Also, elevated Neut# and PLT levels were associated with slower walking speed, consistent with the literature in linking these markers to worse clinical outcomes and increased inflammatory burden [29, 30]. Increased systemic inflammation has been associated with fatigue and impaired recovery [31], which may in turn contribute to decreased walking speed. While prior studies have linked these biomarkers to overall stroke prognosis, our findings suggest that they may also relate specifically to early functional mobility, as captured by walking speed. In addition, increased Neut# levels have been associated with an increased risk of recurrent strokes in mild ischemic stroke patients [32].

In terms of differentiating between mild and severe ischemic stroke using blood analysis results, the literature is limited. WBC, CRP, fibrinogen and erythrocyte sedimentation rates were found to be significantly lower in mAIS compared to moderate to severe stroke measured by NIHSS (mAIS: NIHSS ≤ 5) [33]. Our findings of lower WBC and sodium levels in fast walkers following mAIS confirms these results, however, these effects did not reach statistical significance, likely due to the limited sample size.

Walking speed is an important indicator of long-term outcomes [5] and fall risk [6] following stroke. Decreased walking speed is also associated with all-cause mortality [7, 8] estimating walking speed using readily available blood analytes may have practical implications in the clinics in terms of rehabilitation and fall prevention planning.

Nomograms are commonly utilized for acute ischemic stroke outcome prediction in the literature. The most used outcome measure was dichotomized modified Rankin Scale (mRS) scores. Radiomics features derived from diffusion-weighted imaging images and clinical characteristics such as age, sex, NIHSS scores, along with comorbidities and secondary conditions are widely utilized in the nomograms with AUC values ranging from 80% to 89% [34–38]. Only a few nomograms evaluated blood analytes. Wang et al. and Wu et al. evaluated only the blood sugar levels [34, 39]. Other studies were more comprehensive in terms of blood analytes. Zhang et al. investigated cell counts, CRP, cholesterol, D-dimer and uric acid levels [35]. Zhang et al. evaluated CRP, RDW, HGB, glucose, Na and creatinine levels [38]. Liu et al. considered cell counts, cholesterol, glucose, D-dimer and BUN levels [37]. To the best of our knowledge, this is the first study to investigate a broad panel of routinely analyzed blood analytes to identify candidate features associated with walking speed following mAIS using a nomogram approach.

Similarly, the literature is lacking in terms of nomograms estimating the presence of ischemic stroke based on gait. One prior study incorporated tandem gait test in the development of a nomogram [40]. To our knowledge, this is the first study to include gait speed and smartphone-derived statistical gait features while developing a nomogram for estimating stroke status.

Our findings in the blood analytes nomogram reinforce previous evidence linking renal dysfunction and elevated inflammation (e.g. BUN, eGFR levels and neutrophil counts) to worse clinical outcomes and reduced mobility after ischemic stroke. The nomogram demonstrated discrimination (optimism-corrected AUC = 0.906) comparable to previously reported prognostic models with AUCs between 0.80 and 0.89. The high AUC for gait statistics nomogram (optimism-corrected AUC = 0.878) corroborates the recent findings on the reliability of smartphone inertial sensors for remote stroke monitoring [11, 41]. Nonetheless, the AUC values for both nomograms should be interpreted cautiously given the exploratory nature of the study and the limited sample size.

To address potential confounding by clinical variables, we performed a secondary analysis incorporating age, sex, and NIHSS into both nomograms. In the blood analytes model, the inclusion of sex in the final model resulted in higher model optimism and a decrease in the optimism-corrected AUC from 0.906 to 0.897, justifying the use of the more parsimonious blood-based features. Furthermore, when age was excluded from the blood-based nomogram, optimism-corrected AUC remained relatively strong at 0.743, confirming that blood analytes maintain significant predictive power, independent of age-related frailty or renal burden. Similarly, while NIHSS provided near-perfect discrimination in the gait-based stroke identification model (optimism-corrected AUC = 0.995), its role was tautological as it defines the study groups rather than predicting them, as all control subjects had NIHSS scores of zero. These findings reinforce that the identified signal-based gait statistics and blood analytes provide independent predictive value that is not merely a proxy for baseline clinical severity or demographic factors.

This study shows the feasibility of incorporating the multimodal analysis of blood analytes and gait features into early stroke assessments. Our wearable technology system and the routine nature of these blood analytes make it easier for resource limited areas to utilize this approach.

The primary limitation of this study is the small sample size. The conventional a priori sample size considerations for prediction modeling suggest a minimum of five events per variable. This indicates a requirement for at least 30 outcome events per group for our six-predictor models, making the current state our study underpowered. To address this rigorously, we conducted formal a priori and posteriori power analyses. For the gait-based nomogram, the achieved post-hoc power to detect the observed effect size of the primary predictor (xgyr_max, OR = 0.41) was 0.215, with an a priori analysis indicating that a total sample size of 97 participants would be required to reach the conventional 80% power threshold. The blood-based nomogram demonstrated a post-hoc power of 0.051 for Age (OR = 0.93), with an a priori analysis requiring a sample size of 9511 to achieve 80% power, reflecting the subtle effect size of clinical analytes in this cohort. These results indicate that the current study is underpowered and may be prone to Type II errors. This limitation underscores the necessity of the ridge-penalized regression applied here, which stabilized the regression coefficients and mitigated the risk of overfitting in a limited sample. Accordingly, these findings should be interpreted as exploratory and hypothesis-generating, serving as a preliminary foundation for future multi-center studies that will achieve these calculated sample sizes. Beyond statistical power, the limited sample size may impact model stability and feature selection accuracy; with a high number of candidate features, the models may be sensitive to small variations in the data. We acknowledge that ridge penalization and bootstrap-based internal validation cannot fully eliminate optimism in performance estimates. Finally, the selection of controls from hospital staff and family and friends of the patients may not fully represent the general population, introducing potential selection bias.

We utilized this cohort to develop two nomograms as exploratory tools using internal validation. These tools are not intended for clinical applications without external validation. In the future, we plan to expand the cohort to include stroke mimicking conditions, incorporate external validation and integrate imaging data into our feature set to further explore and validate associations between lesion characteristics, blood analytes and gait features. Methodologically, our approach could be improved by incorporating biomechanically meaningful gait parameters, such as symmetry measures, spatiotemporal features, and step detection-based metrics derived from inertial signals. Future studies may also benefit from validation against reference systems, such as optoelectronic motion capture, to better link signal-derived statistical gait features with established gait constructs. Lastly, we would like to point out that the possible therapeutic power of targeting the inflammatory and kidney blood analytes to improve functional outcome remains to be explored.

## Conclusions

Two ridge-penalized nomograms integrating gait metrics, and renal and inflammatory parameters were developed to estimate stroke status and walking speed in mAIS. These findings show that routine blood analytes and smartphone-based statistical gait features can be combined to differentiate between functional outcomes as measured by walking speed using a nomogram. Analytes of kidney function and inflammation were associated with walking speed. Once validated with external cohorts and larger samples, including stroke-mimicking conditions these features may provide a preliminary framework for determining established blood and gait biomarkers for the detection of mAIS and low walking speed that can help in clinical decision-making particularly in low-resource settings. 

## Supplementary Information

Supplementary Material 1.

## Data Availability

The data used this study are available from the corresponding author, HS, upon reasonable request.

## Abbreviations

AUC: Area under the curve
Baso: Basophils
BUN: Blood urea nitrogen
CI: Confidence Intervals
Cr: Creatinine
CRP: C-reactive protein
eGFR: Estimated glomerular filtration rate
Eos: Eosinophil
HCT: Hematocrit
HGB: Hemoglobin
Lymph: Lymphocyte
Macro-R: Macrocyte ratio
mAIS: Mild acute ischemic stroke
MCH: Mean corpuscular hemoglobin
MCHC: Mean corpuscular hemoglobin concentration
MCV: Mean corpuscular volume
Mono: Monocyte
MPV: Mean platelet volume
Neut: Neutrophil
NIHSS: National Institutes of Health Stroke Scale
NLR: Neutrophil-to-lymphocyte ratio
OR: Odds Ratio
P-LCR: Platelet large cell ratio
PCT: Procalcitonin
PDW: Platelet distribution width
PLT: Platelet count
RBC: Red blood cell count
RDW-CV: Red cell distribution width-coefficient of variation
RDW-SD: Red cell distribution width-standard deviation
TRIPOD: Transparent Reporting of a Multivariable Prediction Model for Individual Prognosis or Diagnosis
TUG: Timed-up-and-go
WBC: White blood cell count
