# Supplementary material for: Identification of signal-based gait features and blood analytes associated with stroke status and walking speed in mild acute ischemic stroke
Source: BMC Neurol. 2026 Jun 12;26:465. doi: 10.1186/s12883-026-05057-3 (PMC13374240; doi:10.1186/s12883-026-05057-3)
Supplement: Supplementary file 1 — Supplementary Material 1. [file 12883_2026_5057_MOESM1_ESM.docx]

**Supplementary Table 1:** Statistical comparison of gait features between control and stroke groups. Data is reported as mean±standard deviation (*: P<0.05, **: P<0.01).

| Features | Control (n=9) (mean±sd) | Stroke (n=22) (mean±sd) | P-value | Features | Control (n=9) (mean±sd) | Stroke (n=22) (mean±sd) | P-value |
| --- | --- | --- | --- | --- | --- | --- | --- |
| CamLeftStance * | 66.27±3.62 | 72.48±10.2 | 0.039 | yacc_std | 1.16±0.21 | 1.06±0.44 | 0.086 |
| CamMeanStance | 67.24±3.6 | 72.08±7.28 | 0.058 | yacc_zerocross | 0.0±0.0 | 0.0±0.0 | 1 |
| CamRightStance | 68.22±3.82 | 71.68±6.04 | 0.064 | ygyr_energy | 77.9±37.87 | 72.8±51.39 | 0.679 |
| CamTUG | 17.15±9.68 | 24.63±13.5 | 0.122 | ygyr_entropy | 3.38±0.17 | 3.41±0.14 | 0.744 |
| CamWalkingSpeed * | 1.02±0.26 | 0.71±0.42 | 0.035 | ygyr_iqr * | 0.26±0.03 | 0.23±0.06 | 0.043 |
| xacc_energy | 163573.27±  89837.93 | 216520.55±  127259.27 | 0.145 | ygyr_kurtosis | 1.14±1.99 | 0.58±0.63 | 0.913 |
| xacc_entropy * | 3.33±0.2 | 3.08±0.3 | 0.018 | ygyr_mad | 0.17±0.03 | 0.14±0.03 | 0.078 |
| xacc_iqr * | 1.7±0.85 | 1.22±1.02 | 0.048 | ygyr_max | 0.74±0.36 | 0.58±0.2 | 0.306 |
| xacc_kurtosis * | 1.76±1.59 | 4.09±2.94 | 0.043 | ygyr_mean | 0.01±0.01 | 0.0±0.01 | 0.286 |
| xacc_mad | 1.18±0.46 | 0.85±0.51 | 0.071 | ygyr_median | 0.01±0.02 | 0.01±0.02 | 0.557 |
| xacc_max | 15.82±2.07 | 15.44±1.95 | 0.811 | ygyr_min | -0.7±0.25 | -0.58±0.16 | 0.133 |
| xacc_mean | 9.58±0.31 | 9.71±0.15 | 0.267 | ygyr_range | 1.44±0.46 | 1.16±0.31 | 0.199 |
| xacc_median | 9.37±0.29 | 9.52±0.22 | 0.103 | ygyr_rms | 0.22±0.04 | 0.18±0.04 | 0.071 |
| xacc_min | 6.25±1.52 | 6.97±1.23 | 0.231 | ygyr_skewness | -0.04±0.76 | -0.04±0.47 | 0.913 |
| xacc_range | 9.57±2.96 | 8.47±2.8 | 0.215 | ygyr_std | 0.22±0.04 | 0.18±0.04 | 0.078 |
| xacc_rms | 9.73±0.35 | 9.8±0.15 | 0.879 | ygyr_zerocross | 0.0±0.0 | 0.0±0.0 | 1 |
| xacc_skewness | 1.03±0.59 | 1.37±0.61 | 0.17 | zacc_energy | 14216.13±  15945.3 | 5613.26±  5166.73 | 0.071 |
| xacc_std | 1.6±0.57 | 1.18±0.61 | 0.086 | zacc_entropy | 3.41±0.14 | 3.4±0.2 | 1 |
| xacc_zerocross | 0.0±0.0 | 0.0±0.0 | 1 | zacc_iqr | 1.54±0.42 | 1.25±0.53 | 0.094 |
| xgyr_energy ** | 664.5±226.03 | 356.83±214.75 | 0.003 | zacc_kurtosis | 0.4±0.77 | 0.12±0.63 | 0.349 |
| xgyr_entropy | 3.26±0.22 | 3.35±0.22 | 0.306 | zacc_mad * | 0.97±0.31 | 0.75±0.34 | 0.048 |
| xgyr_iqr | 0.53±0.18 | 0.45±0.23 | 0.145 | zacc_max | 3.1±2.43 | 3.04±2.7 | 0.811 |
| xgyr_kurtosis | 3.92±3.1 | 2.11±2.62 | 0.122 | zacc_mean | -0.4±2.4 | -0.08±1.47 | 0.17 |
| xgyr_mad * | 0.43±0.12 | 0.31±0.13 | 0.018 | zacc_median | -0.29±2.34 | -0.09±1.4 | 0.184 |
| xgyr_max ** | 2.41±0.92 | 1.37±0.75 | 0.007 | zacc_min | -4.6±3.69 | -3.06±2.16 | 0.071 |
| xgyr_mean ** | 0.14±0.15 | 0.03±0.1 | 0.004 | zacc_range | 7.7±2.46 | 6.1±2.97 | 0.094 |
| xgyr_median | 0.05±0.06 | 0.02±0.05 | 0.071 | zacc_rms ** | 2.52±0.86 | 1.55±0.86 | 0.007 |
| xgyr_min | -1.08±0.94 | -1.1±0.66 | 0.811 | zacc_skewness | -0.3±0.52 | 0.04±0.42 | 0.064 |
| xgyr_range * | 3.49±1.13 | 2.47±0.91 | 0.022 | zacc_std * | 1.25±0.42 | 0.94±0.44 | 0.039 |
| xgyr_rms ** | 0.66±0.2 | 0.43±0.18 | 0.007 | zacc_zerocross | 0.0±0.0 | 0.0±0.0 | 1 |
| xgyr_skewness * | 1.35±1.4 | 0.33±1.29 | 0.022 | zgyr_energy ** | 135.11±67.36 | 71.62±47.93 | 0.007 |
| xgyr_std ** | 0.63±0.2 | 0.42±0.17 | 0.007 | zgyr_entropy | 3.45±0.17 | 3.37±0.17 | 0.215 |
| xgyr_zerocross | 0.0±0.0 | 0.0±0.0 | 1 | zgyr_iqr * | 0.37±0.14 | 0.23±0.11 | 0.031 |
| yacc_energy | 2865.27±  1810.74 | 4089.14±  5123.88 | 0.711 | zgyr_kurtosis | 0.46±0.91 | 0.62±1.0 | 0.811 |
| yacc_entropy | 3.28±0.15 | 3.32±0.26 | 0.286 | zgyr_mad * | 0.22±0.07 | 0.15±0.07 | 0.018 |
| yacc_iqr | 1.49±0.23 | 1.45±0.47 | 0.679 | zgyr_max * | 0.9±0.28 | 0.64±0.27 | 0.018 |
| yacc_kurtosis | 0.68±0.83 | 0.49±1.4 | 0.249 | zgyr_mean | -0.01±0.05 | 0.0±0.01 | 0.184 |
| yacc_mad | 0.91±0.15 | 0.84±0.32 | 0.133 | zgyr_median | -0.0±0.04 | -0.0±0.02 | 0.711 |
| yacc_max | 4.44±1.47 | 3.49±1.88 | 0.094 | zgyr_min * | -0.84±0.34 | -0.6±0.25 | 0.025 |
| yacc_mean | 0.15±0.51 | -0.17±0.86 | 0.327 | zgyr_range * | 1.74±0.6 | 1.24±0.52 | 0.02 |
| yacc_median | 0.09±0.5 | -0.14±0.88 | 0.5 | zgyr_rms * | 0.29±0.09 | 0.19±0.09 | 0.016 |
| yacc_min | -4.15±0.95 | -4.21±2.9 | 0.421 | zgyr_skewness | 0.24±0.28 | 0.08±0.2 | 0.199 |
| yacc_range | 8.59±1.73 | 7.7±4.02 | 0.086 | zgyr_std * | 0.29±0.09 | 0.19±0.09 | 0.02 |
| yacc_rms | 1.28±0.14 | 1.33±0.54 | 0.473 | zgyr_zerocross | 0.0±0.0 | 0.0±0.0 | 1 |
| yacc_skewness | 0.05±0.26 | -0.1±0.22 | 0.133 |  |  |  |  |

**Supplementary Table 2:** Statistical comparison of blood analytes between slow walkers and fast walkers. Data is reported as mean±standard deviation (*: P<0.05, **: P<0.01).

| Features | Slow walkers (n=10) (mean±sd) | Fast walkers (n=12) (mean±sd) | P-value | Features | Slow walkers (n=10) (mean±sd) | Fast walkers (n=12) (mean±sd) | P-value |
| --- | --- | --- | --- | --- | --- | --- | --- |
| Age ** | 78.8±4.96 | 59.67±18.38 | 0.01 | Mono# | 0.8±0.44 | 0.62±0.19 | 0.839 |
| Baso# | 0.05±0.02 | 0.04±0.01 | 0.428 | Mono% | 9.1±2.19 | 8.46±2.61 | 0.678 |
| Baso% | 0.59±0.16 | 0.52±0.13 | 0.325 | MPV | 10.15±0.68 | 10.01±0.5 | 0.418 |
| BUN | 24.7±15.56 | 16.92±2.61 | 0.295 | Na | 139.8±2.3 | 138.92±2.91 | 0.608 |
| Cr | 1.25±0.69 | 0.99±0.1 | 0.641 | Neut# | 5.38±1.61 | 4.38±1.18 | 0.211 |
| eGFR | 54.88±19.37 | 70.07±15.03 | 0.11 | Neut% | 65.03±3.06 | 62.88±6.47 | 0.362 |
| Eos# | 0.25±0.19 | 0.19±0.05 | 0.725 | NIHSS | 2.2±0.79 | 1.83±0.94 | 0.249 |
| Eos% | 3.2±1.87 | 2.84±0.43 | 0.863 | NLR | 3.05±0.67 | 2.66±0.74 | 0.265 |
| HCT | 38.21±4.18 | 38.18±3.95 | 0.973 | P-LCR | 26.04±5.0 | 25.01±3.54 | 0.311 |
| HGB | 12.52±1.6 | 12.75±1.44 | 0.813 | PCT | 0.25±0.03 | 0.22±0.04 | 0.223 |
| K | 4.04±0.17 | 4.11±0.42 | 0.589 | PDW | 11.33±1.34 | 11.1±0.96 | 0.437 |
| Lymph# | 1.81±0.46 | 1.84±0.68 | 0.729 | PLT | 244.9±44.6 | 215.83±38.4 | 0.166 |
| Lymph% | 21.99±3.82 | 25.07±6.32 | 0.265 | RBC | 4.48±0.48 | 4.42±0.34 | 0.813 |
| Macro-R | 3.78±0.34 | 3.68±0.4 | 0.787 | RDW-CV | 14.09±1.01 | 13.67±1.21 | 0.251 |
| MCH * | 28.14±0.97 | 29.26±2.19 | 0.029 | RDW-SD | 44.35±3.09 | 43.96±1.67 | 0.973 |
| MCHC * | 32.73±0.8 | 33.47±0.82 | 0.039 | Urea | 52.38±33.08 | 35.62±5.56 | 0.295 |
| MCV * | 86.14±1.87 | 87.79±4.83 | 0.043 | WBC | 8.37±2.38 | 7.3±1.75 | 0.661 |


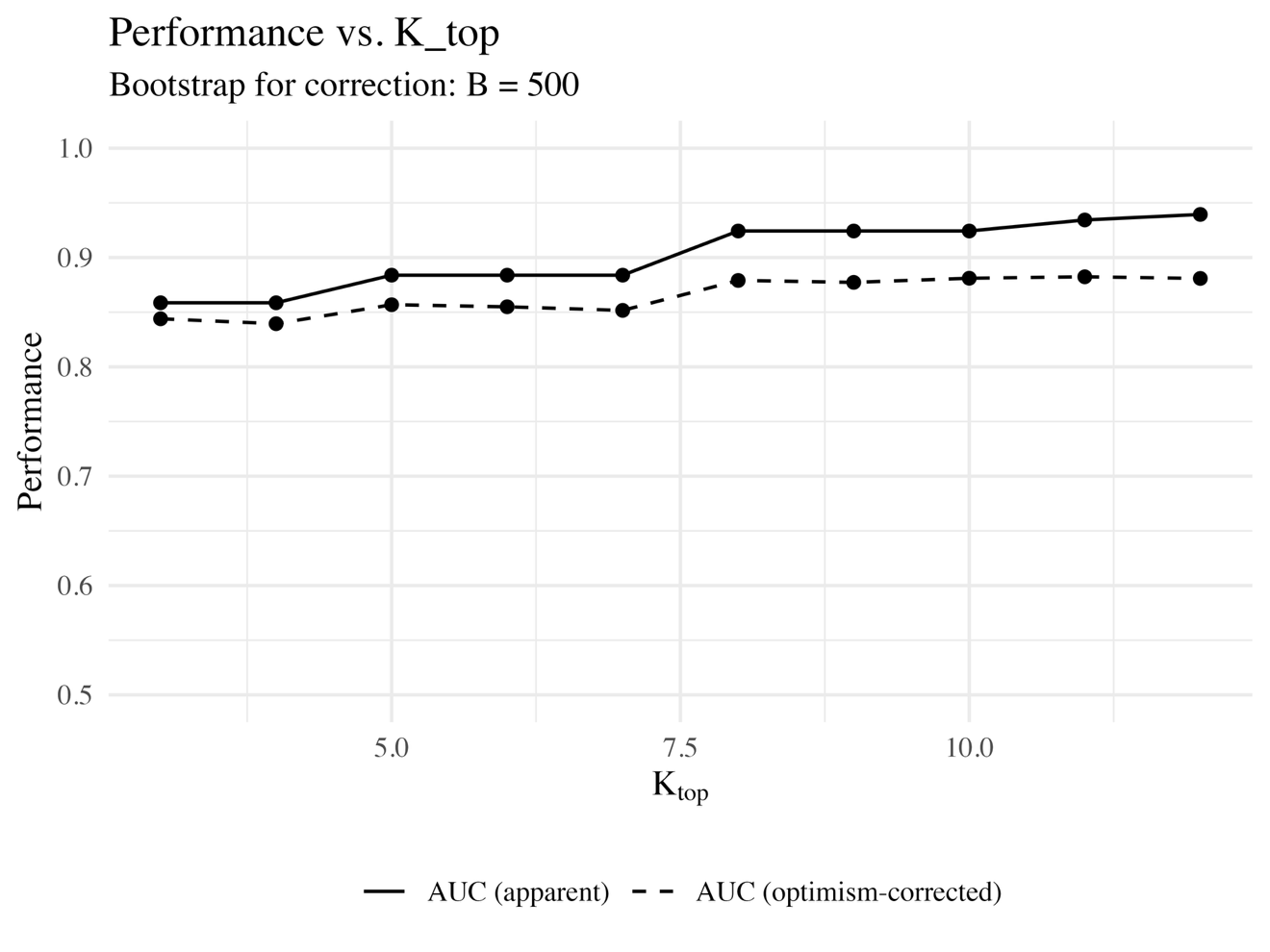


**Supplementary Figure 1:** Performance measured by AUC across candidate feature counts (Ktop). Apparent AUC (solid) and optimism-corrected AUC (dashed) across models with 3–12 gait features.


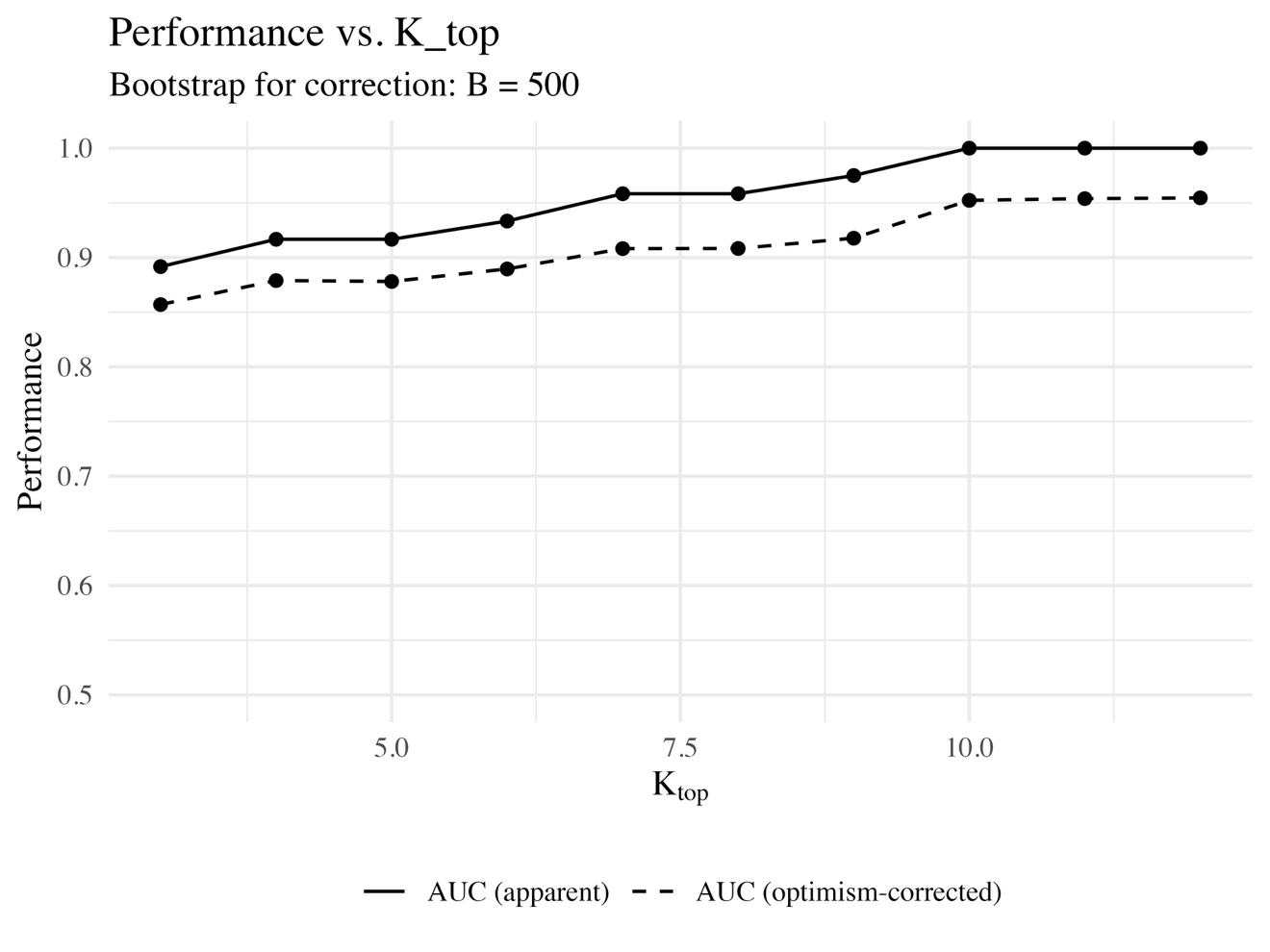


**Supplementary Figure 2:** Performance measured by AUC across candidate feature counts (Ktop). Apparent AUC (solid) and optimism-corrected AUC (dashed) across models with 3–12 blood features.
